# The microstructure of white feathers predicts their visible and near-infrared reflectance properties

**DOI:** 10.1371/journal.pone.0199129

**Published:** 2018-07-05

**Authors:** Devi Stuart-Fox, Elizabeth Newton, Raoul A. Mulder, Liliana D’Alba, Matthew D. Shawkey, Branislav Igic

**Affiliations:** 1 School of BioSciences, The University of Melbourne, Parkville, Victoria, Australia; 2 Department of Biology, Evolution and Optics of Nanostructures group, University of Ghent, Ghent, Belgium; 3 Division of Ecology and Evolution, Research School of Biology, Australian National University, Canberra, Australia; University of Sussex, UNITED KINGDOM

## Abstract

Research on the optical properties of animal integuments, including fur, feather, skin and cuticle, has focussed almost exclusively on animal-visible wavelengths within the narrow range of 300–700 nm. By contrast, the near-infrared (NIR) portion of direct sunlight, spanning 700–2600 nm, has been largely ignored despite its potentially important thermal consequences. We quantified variation in visible and NIR reflectance and transmission for white body contour feathers of 50 bird species, and examined how well they are predicted by feather macro- and micro-structural morphology. Both visible and NIR reflectance of the feathers varied substantially across species. Larger, thicker, and sparser feathers that are characteristic of larger species, and feathers with rounder barbs and more closely spaced barbules, had high average reflectance, particularly within avian-visible wavelengths (300–700 nm). Feathers with rounder barbs and more closely situated barbules also had high average reflectance, particularly for NIR wavelengths. Barb roundness and barbule density were the only predictors of NIR reflectance after accounting for variation in visible reflectance and body size. Our results highlight the potential for adaptive variation in NIR reflectance mediated by feather structure, which may inform the design of functional materials to control light and heat.

## Introduction

Animal integuments reflect sunlight, which comprises ultraviolet (UV, 300–400 nm), human-visible (VIS, 400–700 nm), and near-infrared (NIR, 700–2600 nm) wavelengths, which are invisible to the human eye. Reflection or absorption of solar radiation within NIR wavelengths affect heat gain at an animal’s surface and thus critical thermal limits for survival. This is because surface heat gain depends on the proportion of total incident solar energy that is absorbed or reflected by the body surface (in the absence of convection), and about 55% of the energy in sunlight falls within NIR wavelengths (45% within UV-VIS) [1]. Although NIR reflectance influences the thermal balance of organisms, its potential function in visual communication (e.g. camouflage, communication) are likely minimal or absent (reviewed in [2]). Some animals, such as some snakes, beetles, ticks and mites [3–5] have infrared long wavelength thermal receptors on other parts of the body to sense *heat* (wavelengths > 8000 nm) but these are largely insensitive to the NIR wavelengths of sunlight [5]. NIR light is not visually perceived by most animals, with the possible exception of some fish [6, 7], because photoreceptors with maximum sensitivity (λ_max_) beyond about 630 nm may be too susceptible to thermal noise (i.e. triggered by thermal radiation from sources other than NIR light) [8]. Because NIR wavelengths cannot be seen, selection may separately modulate visual and thermal functions of animal surfaces by acting on visible and NIR properties differently.

NIR reflectance has only been characterised for a few species [9], limiting our understanding of its adaptive significance. We have few empirical data on the relationship between visible and NIR reflectance in animals and the structural components of integuments that influence that relationship. One notable exception is the silver colour of Sahara silver ants (*Cataglyphis bombycina*) that is produced by triangular hairs that reflect much of the sun’s energy and allow the ants to survive in extremely hot conditions [10]. Conversely, the nanoarchitecture of black patches on wings of some butterfly species enhances absorption of visible and NIR radiation [11, 12]. The general lack of data on the structural basis of NIR reflectance properties contrasts dramatically with our extensive understanding of the structural basis of visible colour and ultraviolet. Indeed, a wide range of photonic structures, and their optical effects, have been characterised across organisms including plants, vertebrates, and invertebrates [13]. For example, circularly polarized multilayer reflectors produce green hues in some scarab beetles (reviewed in [14]), whereas “Christmas tree”-like nanostructures produce the iridescent blues on the wings of butterflies in the genus *Morpho* (reviewed in [15]).

Research on animal colouration, especially photonic structures, has had important applications for bio-inspired design of materials that manipulate light. For example, a broad range of optical coatings, optical devices that focus or polarize light, various sensors, and technologies to improve the efficiency of solar cells have all been designed based on photonic structures described from nature [16–18]. Furthermore, the design of fade-resistant and non-toxic “paints” have been influenced by the melanin-based structural colouration of bird feathers [19]. Extending our knowledge of photonic structures to include the NIR may reveal new opportunities for bio-inspired materials that manipulate both light and heat.

Here, we characterise visible and NIR reflectance and transmission spanning the wavelength range 300–2100 nm (encompassing 98.7% of the incident energy of direct sunlight) across white feathers of 50 bird species. We then examined the relationships between these reflectance properties and measurements of feather micro- and macrostructure from Igic *et al*. [20] using a phylogenetic comparative framework. Igic *et al*. [20] characterised the morphological basis of brightness differences across white feathers of 62 bird species. They found that the total visible reflectance of white feathers was predicted by macro- and microstructure of feathers across species. White feathers are of interest because they vary substantially in their perceived brilliance (total reflectance) in the absence of pigments, and because similar structural mechanisms for brilliance of white colours also affect the brilliance of pigment-based feather colours [21, 22]. White is produced by incoherent scattering of light by disordered nanostructures [23–25]. Our study aimed to determine how such structures affect NIR reflectance and its relationship to visible reflectance.

## Methods

### Sample selection and general approach

We used white feathers of 50 of the species in Igic *et al*. [20], and their associated measurements of feather macro- and microstructure. Due to specimen availability and export restrictions, we could not include 12 specimens from Igic *et al*. [20] in the current study. We measured reflectance and transmission, from 300 to 2100 nm, of unpigmented contour feathers from head and body (mostly breast and abdomen) patches from 50 species at the University of Akron Ornithological Collection (S1 Table). Species were chosen to represent size and ecological diversity of birds, as size and ecology (e.g aquatic habit) may influence feather structure [20]. Three feathers were obtained from each specimen per species and sent to the University of Melbourne for spectral measurements. Feathers were cleaned using ethanol (sensu [20]) to remove dirt and other potential contaminants.

### Spectral measurements

We measured specular reflectance and transmission of the pennaceous regions of whole feathers across 300–2100 nm. This spectral range encompasses 98.7% of the total incident solar radiation, assuming the ASTM G-173-03 standard irradiance spectrum for dry air [26]. Spectral measurements were taken with an Ocean Optic dual-spectrometer system (Ocean Optics, Inc. USA) consisting of two spectrometers (USB2000+ [300–1000 nm] and NIRQuest [1000–2100 nm]) with two light sources (PX-2 pulsed Xenon light for the UV-visible range and HL-2000 tungsten halogen lights for the visible-NIR range) connected with a quadrifurcated fibre optic (Ocean Optics, 2m, 600 μm diameter). Measurements were calibrated against a Spectralon 99% diffuse reflectance standard (Labsphere, USA).

Feathers were measured on a black aluminium foil background (GAM BlackWrap, GAMProducts Inc., USA), which has low reflectance (< 5%) across 300–2100 nm. We used BlackWrap rather than black velvet because the reflectance of black velvet rises sharply in the NIR, rather than being uniformly low across all wavelengths of interest. We stacked 3 feathers (depending on availability) to minimise transmission, which was particularly prominent for small, fine feathers. Three measurements were taken for each species on the dorsal side of the stacked feathers; one on either side of the rachis and one on the best side of the rachis in terms of feather quality (e.g. avoiding frayed feathers with unaligned barbules), except when feathers were too small to exclude the rachis. Reflectance measurements were taken using an anodised aluminium probe holder with an ovoid aperture of 4 x 3 mm at coincident oblique (45°) geometry.

Transmission was measured through the pennaceous region of a single feather, repeated on 1–3 feathers per species. Measurements were made with the same dual spectrometer system as above but with separate fibre optics for the illuminant and reflectance probe, each of which had a 74-series collimating lens attached, mounted in a 74-ACH collimating lens holder (Ocean Optics Inc., USA) set 40 mm apart. Feathers were oriented so that light passed through the feather from its dorsal side. Feathers were positioned so the light passed through barbs and barbules while avoiding the rachis, except for feathers that were smaller than the beam of light.

**Fig 1:**
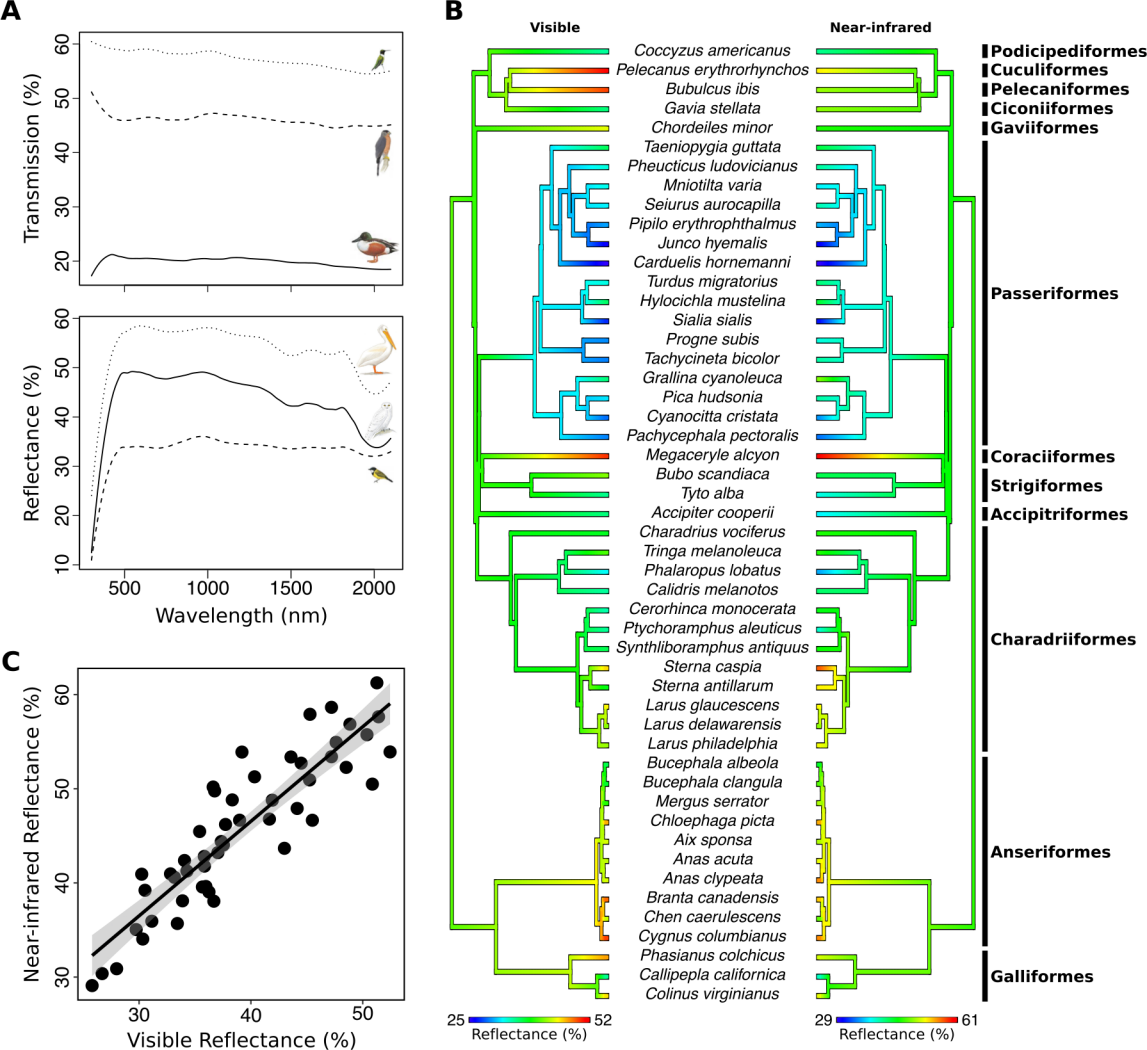
Variation in average avian visible (300–700 nm) and average near-infrared (700–2100 nm) reflectance and transmission of white body contour feathers. (a) Representative transmission (top) and reflectance spectra (bottom) for species in this study (top to bottom: *Archilochus colubrisi*, ruby-throated hummingbird; *Accipiter cooperii*, Cooper’s hawk; *Anas clypeata*, northern shoveler; *Pelecanus erythrorhynchos*, American white pelican; *Bubo scandiacus*, snowy owl; *Pachycephala pectoralis*, golden whistler). (b) Dendrograms showing phylogenetic distributions of average avian visible (left) and average near-infrared reflectance. (c) Relationship between average avian visible reflectance and average near-infrared reflectance.

We averaged the three reflectance scans for each species to give a species’ mean reflectance spectrum. We then calculated the average reflectance across the visible (300–700 nm) and NIR (700–2100 nm) ranges. As visible and NIR reflectance are correlated (see Results), we calculated NIR reflectance adjusted for visible reflectance (relative NIR reflectance) by regressing NIR reflectance against visible reflectance and extracting the residuals. As transmission was consistent (broadband and flat) throughout the UV-Vis and NIR ranges (Fig 1B), we used total average transmission (300–2100 nm).

We assessed how reflectance variation might influence the heat load at a bird’s surface by calculating the net radiation gain for an animal exposed to direct sunlight, assuming constant solar intensity of 1103 W m^-2^ s^-2^. Forty-five percent of this solar radiation (0.45 x 1103 W m^-2^ s^-2^ = 496.35 W m^-2^ s^-2^) falls within visible wavelengths (300–700 nm) and the other 55% (0.55 x 1103 W m^-2^ s^-2^ = 606.65 W m^-2^ s^-2^) falls within NIR wavelengths (700–2100 nm). Thus, heat load at the surface can be estimated as [(1 –R_vis_) x 496.35 W m^-2^ s^-2^] + [(1 –R_NIR_) x 606.65 W m^-2^ s^-2^], where R refers to % reflectance [2]. Values are meant to be indicative only as this simplified equation omits effects of all other sources of radiation apart from direct sunlight (e.g. sunlight reflected off other surfaces and thermal (long-wave) radiation).

### Feather morphology

**Fig 2:**
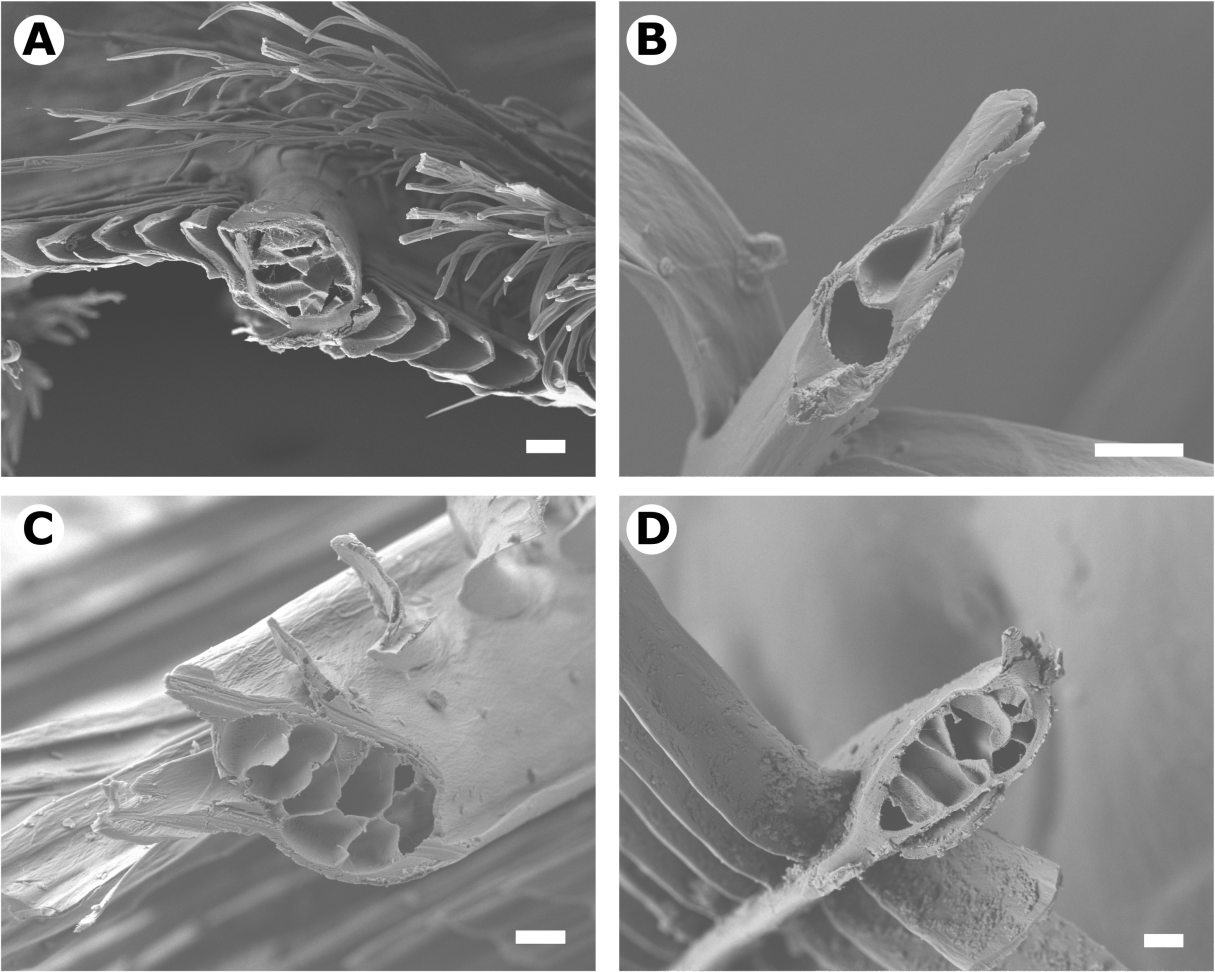
Morphological variation of white feather barbs. Scanning electron micrographs of cross-sections through barbs of feathers with the high (a) and low (b) average near-infrared reflectance or high (c) and low (d) relative NIR reflectance after accounting for variation in avian visible reflectance. (a) *Megaceryle alcyon*, belted kingfisher; (b) *Acanthis hornemanni*, arctic redpoll; (c) *Grallina cyanoleuca*, magpie-lark; (d) *Bubo scandiacus*, snowy owl. Scale: 10 μm. SEM data and images are from Igic *et al*. [20]. Images were created using a JSM-7401F SEM (JEOL, Tokyo, Japan), at a working distance of 7 mm, and an accelerating voltage of 7 kV.

Methods for characterising feather morphology are detailed in Igic *et al*. [20]. Briefly, 11 feather structure characteristics were measured from light and scanning electron microscopy images (Fig 2; S1 Fig): (i) barb size as the cross-sectional surface area of barbs; (ii) barb aspect ratio as the cross-sectional dorso-ventral length relative to lateral length; (iii) size of barb medullary layer as a proportion of barb size; (iv) proportion of the barb medullary layer that is composed of air pockets and the (v) total perimeter of these air pockets, which collectively approximated the size and uniformity of air gaps within the barb; (vi) barbule size as a residual from a linear regression of barbule surface area against the barbules distance from the barb (negative and positive respectively indicate barbules that are smaller or larger than average); (vii) number of medullary layer compartments on barb cross-sections; (viii) barb sparsity as the number of barbs per 1 mm rachis length; (ix) barbule sparsity as the number of barbules per 1 mm barb length; (x) barbule length as the longitudinal length of barbules; and (xi) rachis length as the longitudinal length of the rachis.

To improve linearity of variables, prior to analyses we log-transformed barb thickness, barb sparsity, and barb aspect ratio measurements, and arcsine-transformed all proportion measurements as in Igic *et al*. [20]. We then conducted a principal component analysis (PCA) on the 11 feather characteristics.

### Comparative analyses

We used phylogenetic linear regression to test for relationships between reflectance or transmission features of whole feathers and the principal components derived from the feather structure measurements. We ran four models with the response variables of average visible reflectance (300–700 nm), average NIR reflectance (700–2100 nm), relative NIR reflectance (residuals of NIR on visible reflectance), or average total transmission (300–2100 nm). In each model, we included PC1, PC2, and PC3 (see Results). We ran models with and without log body mass because it was strongly correlated with feather structure (PC1 and PC3 see Results). The log average species body mass from Handbook of Birds of the World [27] was used as an approximation of body size differences across species. The amount of phylogenetic non-independence between species was accounted for by simultaneously computing a measure of phylogenetic signal in the residuals of the models using Pagel’s λ [28, 29]. To account for phylogenetic uncertainty, we repeated the analysis across 100 iterations of Hackett backbone phylogenetic trees downloaded from birdtree.org [30] and calculated average estimates and standard errors. Analyses were performed in R version 3.2.2. (R core team) using the packages phytools [31] and phylolm [32].

Data were checked for normality and outliers, and R^2^ values for models were calculated, using the pgls() function of the caper package [33] and by inspecting reflectance spectra manually. Variance inflation factors of the variables in the models were calculated using the vif() function of the package car [34] and the gls() function of the package nlme [35].

## Results

### Variation in reflectance properties and feather morphology among species

Both visible and NIR reflectance spectra for white feather patches were similar in shape across species but varied substantially in their mean values (average % reflectance over the relevant wavelength range; Fig 1A). Average visible reflectance ranged from 25.8% for the hoary redpoll, *Carduelis hornemanni*, to 52.4% for the American white pelican, *Pelecanus erythrorhynchos*, whereas average NIR reflectance ranged from 29.1% for the hoary redpoll, *Carduelis hornemanni*, to 61.3% for the belted kingfisher, *Megaceryle alcyo*n.

Although average visible and NIR reflectance were highly positively correlated (Table 1, Fig 1C), there was approximately 14% variation in average NIR reflectance after accounting for visible reflectance (residuals of average % visible reflectance regressed again average % NIR reflectance: range = -6.6–8.26%). Relative NIR reflectance was lowest for the cattle egret, *Bubulcus ibis* (-6.6% average relative NIR) and highest for the least tern, *Sterna antillarum* (8.26%). Furthermore, relative NIR reflectance was positively correlated with average NIR reflectance (Table 1). As an indication of how this may affect energy gain at the feather surface when exposed to direct sunlight, estimated energy gain is 543 W m^-2^ s^-2^ for the cattle egret, *Bubulcus ibis*, and 413 W m^-2^ s^-2^ for the belted kingfisher, *Megaceryle alcyon*. This energy gain difference of 31% was related to differences in the species’ average NIR reflectance (50.5% and 61.25% respectively); both species had average visible reflectance of 51%.

**Table 1:** Pairwise correlations among phylogenetically independent contrasts of feather average reflectance and transmission properties (N = 50).

	Visible refl.	NIR refl.	Relative NIR	Total transm.
Visible refl.	-	**<0.0001**	0.26	**0.0007**
NIR refl.	**0.88**	-	**<0.0001**	**<0.0001**
Relative NIR	0.16	**0.62**	-	**<0.0001**
Total transm.	**-0.47**	**-0.66**	**-0.58**	-

P-values are above the diagonal and Pearson correlation coefficients are below the diagonal. Significant relationships after False Discovery Rate correction for multiple tests are in bold (α = 0.008).

Transmission was similar across wavelengths, with a slight decrease at longer wavelengths (Fig 1A). Total transmission across species ranged from 15–60%. As expected when absorption is negligible, average total transmission was negatively correlated with both visible and NIR reflectance (Table 1).

The first three principal components of feather morphology together accounted for 72% of the variation in feather microstructure across species (Table 2). Species with larger, thicker, and sparser feathers (larger spaces between barbs and barbules) had larger positive scores on PC1. Unsurprisingly, PC1 was strongly associated with body mass (Table 3) because larger species had larger, thicker, and sparser feathers. High values of PC2 indicate more internally complex feathers (relatively larger medullary layer with smaller air pockets), more closely situated barbs and smaller barbules (Table 2). High values of PC3 indicate feathers with rounder barb shapes and much more closely situated barbules (Table 2).

**Table 2:** Eigenvectors and eigenvalues of the first three principal components (PC) from a PCA of 11 feather morphology measurements for 50 bird species.

	Measurement	PC1	PC2	PC3
(i)	Barb size	0.433	0.188	0.278
(ii)	Barb aspect-ratio	0.14	-0.132	-0.581
(iii)	Barb medullary size	0.16	0.452	0.072
(iv)	Size of barb medullary layer air pockets	0.065	-0.274	0.148
(v)	Perimeter of barb medullary layer air pockets	0.423	0.204	0.3
(vi)	Barbule size	0.177	-0.457	0.211
(vii)	Number of barb medullary layer compartments	0.241	0.42	-0.105
(viii)	Barb sparsity	-0.301	0.332	0.255
(ix)	Barbule sparsity	-0.137	-0.256	0.574
(x)	Barbule length	0.422	-0.189	-0.135
(xi)	Rachis length	0.456	-0.174	0.029
	**Eigenvalue**	3.554	2.917	1.454
	**Proportion of variance**	0.323	0.265	0.132
	**Cumulative proportion**	0.323	0.588	0.72

**Table 3:** Effect of feather structure (PC1, PC2, and PC3) on the visible and NIR reflectance and transmission of white feathers, and on body mass. Estimate (95% CI) refers to the average slope and 95% confidence intervals for analyses run over 100 trees.

	Effect	Estimate (95% CI)	t	P
Visible reflectance				
	Intercept	38.06 (36.06, 40.06)	38.33	<0.0001
	Body part	6.35 (1.00, 11.70)	2.39	0.02
	PC1	2.17 (1.15, 3.20)	4.25	0.0001
	PC2	1.66 (-0.42, 3.76)	1.60	0.12
	PC3	2.93 (1.54, 4.32)	4.23	0.0001
	Lambda (95% CI)	0.074 (0.04, 0.11)		
	R squared	0.53		
NIR reflectance				
	Intercept	44.85 (42.86, 46.84)	45.30	<0.0001
	Body part	1.90 (-3.95, 7.76)	0.65	0.52
	PC1	1.43 (0.31, 2.56)	2.56	0.014
	PC2	2.44 (0.10, 4.79)	2.10	0.04
	PC3	4.23 (0.75, 5.73)	5.66	<0.0001
	Lambda (95% CI)	0.022 (0.032, 0.045)		
	R squared	0.58		
Relative NIR				
	Intercept	0.32 (-2.34, 2.99)	0.24	0.81
	Body part	-5.26 (-9.34, -1.19)	-2.60	0.01
	PC1	-0.79 (-1.47, -0.10)	-2.31	0.03
	PC2	0.89 (-0.34, 2.09)	1.45	0.15
	PC3	1.33 (0.36, 2.31)	2.74	0.0085
	Lambda (95% CI)	0.65 (0.54, 0.72)		
	R squared	0.33		
total transmission				
	Intercept	31.09 (21.57, 40.61)	6.56	<0.0001
	Body part	11.97 (-0.40, 24.33)	1.95	0.06
	PC1	0.56 (-1.22, 2.34)	0.63	0.53
	PC2	-3.55 (-6.48, -0.62)	-2.43	0.02
	PC3	-2.84 (-5.31, -0.37)	-2.32	0.02
	Lambda (95% CI)	0.86 (0.81, 1.0)		
	R squared	0.30		
Log body mass				
	Intercept	2.34 (1.90, 2.77)	10.80	<0.0001
	Body part	0.24 (-0.31, 0.79)	0.89	0.38
	PC1	0.28 (0.20, 0.35)	7.11	<0.0001
	PC2	-0.12 (-0.25, 0.006)	-1.91	0.06
	PC3	0.12 (0.01, 0.23)	2.23	0.03
	Lambda (95% CI)	0.93 (0.88, 0.95)		
	R squared	0.55		

### Relationships between feather reflectance, transmission and morphology

**Fig 3:**
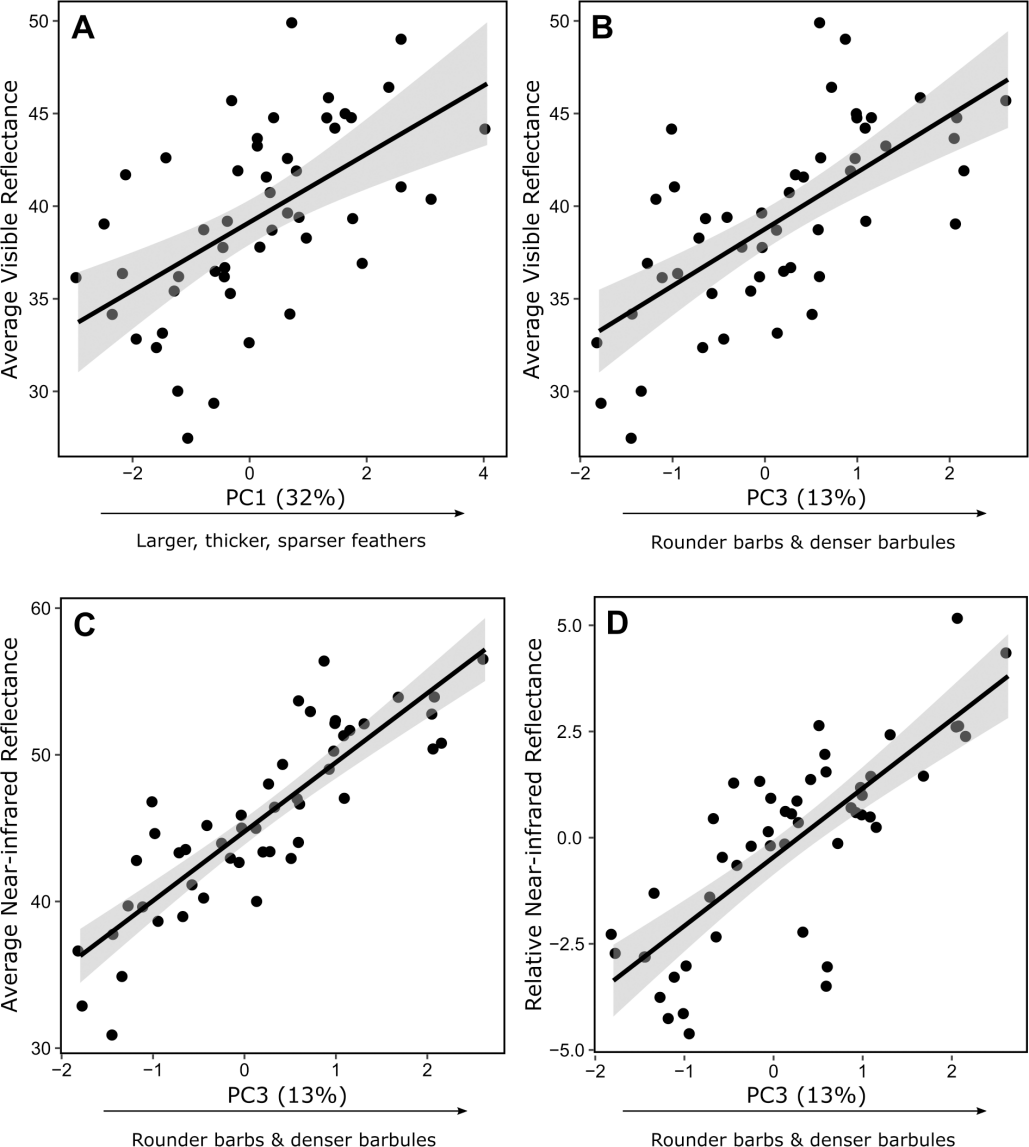
Relationships between feather reflectance and structure. (a) Average avian visible reflectance in respect to feather size and thickness (PC1). (b) Average avian visible reflectance, (c) average NIR reflectance, and (d) relative NIR reflectance. Values are those predicted from the model and thus show reflectance in respect to the selected PCs while keeping differences in other PCs constant and accounting for phylogeny. Percentages illustrate the proportion of total variance in feather structure explained by principal components.

Visible and NIR reflectance was predicted by feather structure. Species with higher visible reflectance had larger, thicker, sparser feathers (positive association with PC1) and rounder barbs with more tightly packed barbules (positive association with PC3; Table 3; Fig 2; Fig 3A and 3B). Our feather structure measurements explained 55% of the visible reflectance variation across species (Table 3). Average and relative NIR reflectance were strongly predicted by feather microstructure (PC3): both increased with barb roundness and more closely situated barbules (Fig 2; Fig 3C and 3D). Average NIR reflectance was also positively but weakly associated with other aspects of feather structure, namely larger, thicker, sparser feathers (PC1), and more internally complex feathers with more closely situated barbs and smaller barbules (PC2). However, relative NIR reflectance was weakly negatively associated with PC1 and not significantly associated with PC2. Thus, barb roundness and barbule density (PC3) are the strongest predictors of NIR reflectance independent of visible reflectance. Our feather structure measurements respectively explained 58% and 33% of variation across species’ average and relative NIR reflectance (Table 3).

Transmission was negatively associated with both PC2 and PC3 (Table 3), indicating that light transmitted better through feathers with less hollow and flatter barbs, less medullary region and thicker but sparser barbules.

### Feather reflectance and body size

Average visible and NIR reflectance were both predicted by body size (S2 Table). Because of the strong correlation between body size and PC1 (larger species had larger, thicker and sparser feathers), PC1 did not predict average visible reflectance when body size was included in the model (S2 Table). However, visible and NIR and relative NIR reflectance were still positively associated with barb roundness and barbule density (PC3) when accounting for body size differences (S2 Table). Average visible and NIR reflectance were also positively associated with PC2, and transmission negatively associated with PC2 (S2 Table). Species with higher average visible and NIR reflectance, or lower total transmission, had more internally complex feathers (relatively larger medullary layer with more, but smaller, air pockets), more closely situated barbs, and smaller barbules.

## Discussion

A condition for the adaptive value of NIR reflectance is that it varies significantly among species. We demonstrated that NIR reflectance of white feathers varies across bird species and is predicted by feather structural differences. Both average visible and NIR reflectance of white feathers varied by about 30%, and although visible and NIR reflectance were strongly correlated, NIR reflectance varied substantially after accounting for visible reflectance. The cattle egret and the snowy owl, *Bubo scandiacus*, showed low NIR relative to visible reflectance, whereas the magpie lark, *Grallina cyanoleuca*, red-throated loon, *Gavia stellata*, and least tern, *Sternula antillarum*, showed relatively high NIR reflectance. This variation in NIR reflectance has the potential to influence surface heat gain, and thus the thermal properties of white feathers.

Both visible and NIR reflectance were predicted by structural properties of feathers. Visible reflectance (brilliance or brightness) increased strongly with increasing feather size and thickness, and more closely spaced barbs and barbules. The relationship between white brilliance and feather size and thickness is primarily driven by body size (larger species have larger, thicker feathers) and disappears when variation in body size is accounted for. However, species with rounder barbs and more closely spaced barbules had higher visible and NIR reflectance, even after accounting for body size differences. These two aspects of feather structure (barb roundness and barbule density) had a greater effect on NIR than visible reflectance, and therefore also predicted relative NIR. Nevertheless, as 33–58% of the variation in visible and NIR reflectance across species was explained by our models, after accounting for phylogeny, other structural features of feathers that were not measured here may also contribute to their reflectance differences.

Igic *et al’s*. [20] analysis of diffuse and specular reflectance of single feathers across the avian-visible range (300–700 nm) showed that feather brilliance is associated with greater feather volume, achieved through either greater barb thickness and internal complexity or more closely packed barbs and barbules. This is likely because complex internal nanostructures, and thicker and denser barbs and barbules, increase the scattering of light. Similarly, higher visible and NIR reflectance of stacked feathers in our study was associated with rounder barbs and more closely packed barbules, after accounting for feather size and thickness. Thus, unsurprisingly, greater surface area for light scattering and reflection increases both visible and NIR reflectance.

Although visible reflectance, including the brilliance of white feathers, is primarily driven by selection for signalling functions [36–40], NIR reflectance is presumably driven by selection on thermoregulatory processes. Based on our calculations, bird species with similar visible reflectance could experience a 30% difference in heat loads at the surface due specifically to differences in their NIR reflectance. However, whether this translates to heat load at the skin (which is what is relevant to the animal’s thermal balance) depends on the thickness and optical properties of the insulating feather layer. In any case, the difference in heat load at the skin would be less than 30% because the insulating layer will attenuate radiation reaching the skin. A combination of biophysical models and experimental data are required to determine how variation in NIR reflectance influences biologically relevant thermal properties.

Mimicking nanostructures of natural materials with useful or interesting properties such as hydrophilic lotus leaves [41] and iridescent feathers [19] has led to both fundamental insights and development of novel functional materials. However, relatively few coatings are designed to specifically affect both visible and NIR wavelengths, and most are metal-based, with mirror-like appearances [42–44]. White materials have more practical applications, and in the case of feathers are made from more inexpensive and biodegradable materials than metals (e.g. β-keratin [45]). Moreover, our finding that materials with the same visible brightness vary in NIR reflectance means that identically-bright coatings could potentially be tailored to suit different thermal environments, i.e. more reflective in hot environments and less reflective in others. This possibility is clearly far from being implemented [46], but seeing that it is possible may help inspire the next generation of materials that manipulate both light and heat.

Overall, our results suggest that selection could operate on different aspects of feather structure to manipulate visible and NIR reflectance properties for competing functions (e.g. signalling and camouflage *versus* thermoregulation). The potential adaptive significance of NIR reflectance is clearly mediated by body size because larger species have a thicker thermal boundary layer and higher thermal lag, leading to the common assumption that larger animals are ‘radiation coupled’ whereas smaller animals are ‘convection coupled’ [1]. Our data show that larger species have higher visible and NIR reflectance for white feathers, due to the size-coupled structure of their feathers. However, small species may be able to make rapid temperature adjustments by adjusting behaviour due to the high transmission of light through their feathers [47]. It remains to be seen whether variation in NIR reflectance has evolved to provide thermal benefits in birds. However, our data confirm that the structural properties of feathers—specifically those that influence surface area and reflection—can generate substantial variation in NIR reflectance properties, which influence surface radiative heat gain.

## Supporting information

S1 Fig Summary of measurements taken on feather structure. Figure reproduced with permission from Igic *et al*. (2018).
(TIF)

S1 Table Full species list and any associated information. (DOCX)

S2 Table Effect of feather structure (PC1, PC2, and PC3) and log body mass on the visible and NIR reflectance and transmission of white feathers. (DOCX)
