# Supplementary figures and images for: The microstructure of white feathers predicts their visible and near-infrared reflectance properties
Source: PLoS One. 2018 Jul 5;13(7):e0199129. doi: 10.1371/journal.pone.0199129 (PMC6033395; doi:10.1371/journal.pone.0199129)

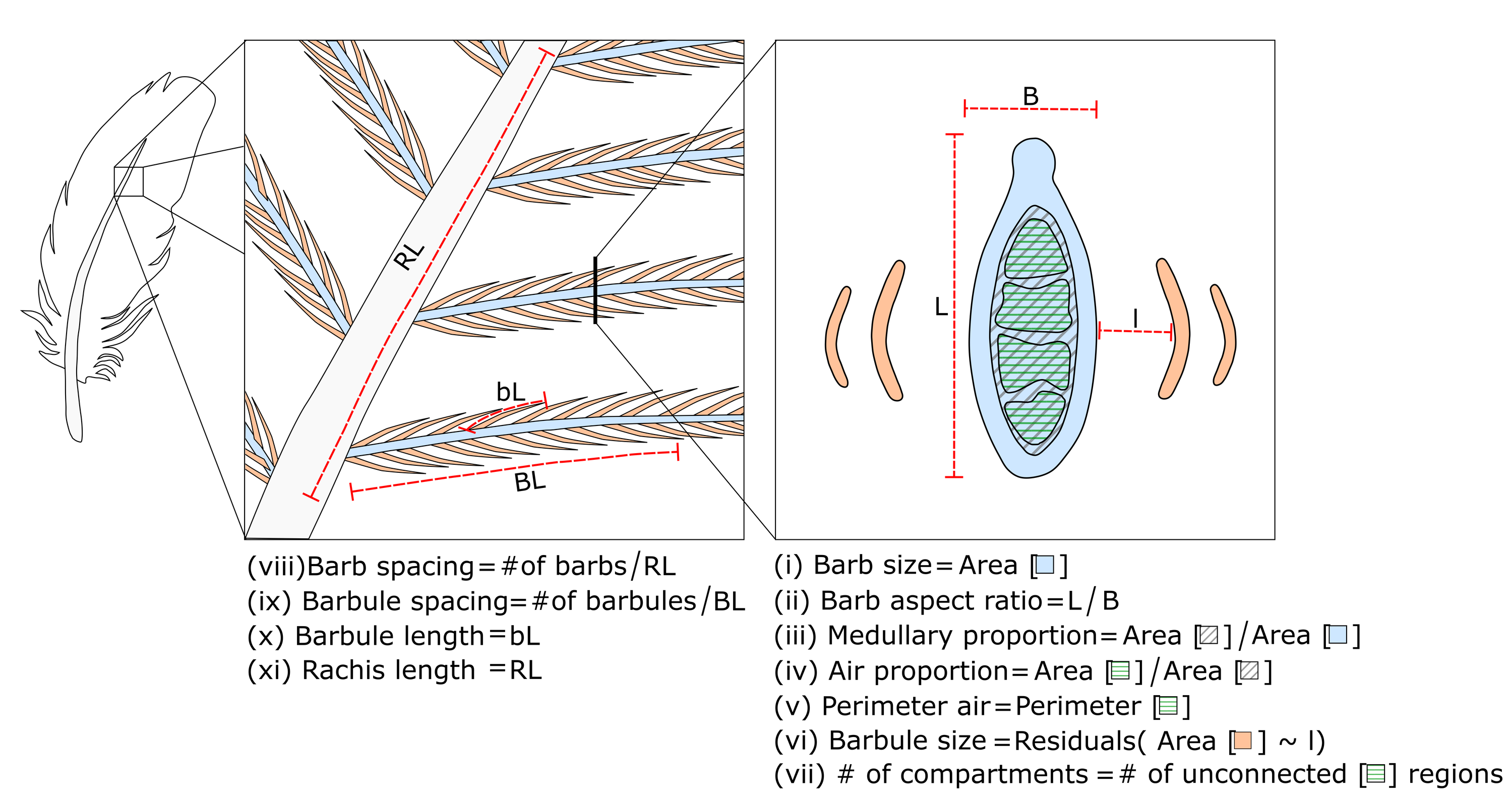

Supplement: S1 Fig — Figure reproduced with permission from Igic et al. (2018). (TIF) [file pone.0199129.s001.tif]
