# Supplementary material for: The microstructure of white feathers predicts their visible and near-infrared reflectance properties
Source: PLoS One. 2018 Jul 5;13(7):e0199129. doi: 10.1371/journal.pone.0199129 (PMC6033395; doi:10.1371/journal.pone.0199129)
Supplement: S1 Table — (DOCX) [file pone.0199129.s002.docx]

**S1 Table.** Full species list and any associated information.

| **Order** | **Family** | **Latin name** | **Body part** | **Sex** | **Collection Year** |
| --- | --- | --- | --- | --- | --- |
| Accipitriformes | Accipitridae | *Accipiter cooperii* | body | unknown | unknown |
| Anseriformes | Anatidae | *Aix sponsa* | body | male | unknown |
| Anseriformes | Anatidae | *Anas acuta* | body | male | unknown |
| Anseriformes | Anatidae | *Anas clypeata* | body | male | 1982 |
| Anseriformes | Anatidae | *Branta canadensis* | body | female | unknown |
| Anseriformes | Anatidae | *Bucephala albeola* | body | female | unknown |
| Anseriformes | Anatidae | *Bucephala clangula* | body | male | unknown |
| Anseriformes | Anatidae | *Chen caerulescens* | body | male | unknown |
| Anseriformes | Anatidae | *Chloephaga picta* | body | male | unknown |
| Anseriformes | Anatidae | *Cygnus columbianus* | body | female | 1977 |
| Anseriformes | Anatidae | *Mergus serrator* | body | unknown | unknown |
| Caprimulgiformes | Caprimulgidae | *Chordeiles minor* | head | male | 1981 |
| Charadriiformes | Alcidae | *Cerorhinca monocerata* | body | female | 1992 |
| Charadriiformes | Alcidae | *Ptychoramphus aleuticus* | body | female | 1997 |
| Charadriiformes | Alcidae | *Synthliboramphus antiquus* | body | female | 1997 |
| Charadriiformes | Charadriidae | *Charadrius vociferus* | body | unknown | unknown |
| Charadriiformes | Laridae | *Larus delawarensis* | body | male | 1989 |
| Charadriiformes | Laridae | *Larus glaucescens* | body | male | 1987 |
| Charadriiformes | Laridae | *Larus philadelphia* | body | male | 1978 |
| Charadriiformes | Scolopacidae | *Calidris melanotos* | body | unknown | unknown |
| Charadriiformes | Scolopacidae | *Phalaropus lobatus* | body | unknown | unknown |
| Charadriiformes | Scolopacidae | *Tringa melanoleuca* | body | unknown | unknown |
| Charadriiformes | Sternidae | *Hydroprogne caspia* | body | unknown | 1982 |
| Charadriiformes | Sternidae | *Sterna antillarum* | body | unknown | unknown |
| Ciconiiformes | Ardeidae | *Bubulcus ibis* | body | male | unknown |
| Coraciiformes | Cerylidae | *Megaceryle alcyon* | body | unknown | unknown |
| Cuculiformes | Cuculidae | *Coccyzus americanus* | body | male | unknown |
| Galliformes | Odontophoridae | *Callipepla californica* | head | male | 1982 |
| Galliformes | Odontophoridae | *Colinus virginianus* | head | male | unknown |
| Galliformes | Phasianidae | *Phasianus colchicus* | head | male | 1983 |
| Gaviiformes | Gaviidae | *Gavia stellata* | body | unknown | unknown |
| Passeriformes | Cardinalidae | *Pheucticus ludovicianus* | body | male | unknown |
| Passeriformes | Corvidae | *Cyanocitta cristata* | body | female | unknown |
| Passeriformes | Corvidae | *Pica hudsonia* | body | female | unknown |
| Passeriformes | Emberizidae | *Junco hyemalis* | body | male | unknown |
| Passeriformes | Emberizidae | *Pipilo erythrophthalmus* | body | male | unknown |
| Passeriformes | Estrildidae | *Taeniopygia guttata* | body | male | 1986 |
| Passeriformes | Fringillidae | *Acanthis hornemanni* | body | unknown | unknown |
| Passeriformes | Hirundinidae | *Progne subis* | body | female | unknown |
| Passeriformes | Hirundinidae | *Tachycineta bicolor* | body | female | unknown |
| Passeriformes | Monarchidae | *Grallina cyanoleuca* | body | male | unknown |
| Passeriformes | Pachycephalidae | *Pachycephala pectoralis* | head | male | unknown |
| Passeriformes | Parulidae | *Mniotilta varia* | body | male | unknown |
| Passeriformes | Parulidae | *Seiurus aurocapilla* | body | female | unknown |
| Passeriformes | Turdidae | *Hylocichla mustelina* | body | male | unknown |
| Passeriformes | Turdidae | *Sialia sialis* | body | unknown | unknown |
| Passeriformes | Turdidae | *Turdus migratorius* | body | male | 1976 |
| Pelecaniformes | Pelecanidae | *Pelecanus erythrorhynchos* | body | female | unknown |
| Strigiformes | Strigidae | *Bubo scandiaca* | body | unknown | unknown |
| Strigiformes | Tytonidae | *Tyto alba* | body | unknown | unknown |
