# Supplementary material for: The microstructure of white feathers predicts their visible and near-infrared reflectance properties
Source: PLoS One. 2018 Jul 5;13(7):e0199129. doi: 10.1371/journal.pone.0199129 (PMC6033395; doi:10.1371/journal.pone.0199129)
Supplement: S2 Table — (DOCX) [file pone.0199129.s003.docx]

**S2 Table.** Effect of feather structure (PC1, PC2, and PC3) and log body mass on the visible and NIR reflectance and transmission of white feathers.

|  | Effect | Estimate (95% CI) | t | P |
| --- | --- | --- | --- | --- |
| Visible reflectance | |  |  |  |
| Intercept | 28.71 (22.59, 34.84) | 9.43 | <0.0001 |  |
|  | Body part | 3.97 (-1.19, 9.19) | 1.55 | 0.13 |
|  | PC1 | 0.72 (-0.71, 2.14) | 1.01 | 0.32 |
|  | PC2 | 2.0 (0.03, 3.97) | 2.04 | 0.05 |
|  | PC3 | 1.84 (0.33, 3.35) | 2.45 | 0.018 |
|  | Mass | 4.25 (1.43, 7.06) | 3.03 | 0.004 |
|  | Lambda (95% CI) | <0.01 (<0.01, <0.01) |  |  |
|  | R squared | 0.68 |  |  |
| NIR reflectance | |  |  |  |
|  | Intercept | 34.82 (28.00, 41.64) | 10.26 | <0.0001 |
|  | Body part | -1.06 (-6.85, 4.72) | -0.37 | 0.71 |
|  | PC1 | -0.27 (-1.86, 1.32) | -0.34 | 0.71 |
|  | PC2 | 2.81 (0.62, 5.0) | 2.58 | 0.013 |
|  | PC3 | 2.86 (1.18, 4.54) | 3.42 | 0.001 |
|  | Mass | 4.67 (1.54, 7.81) | 3.0 | 0.004 |
|  | Lambda (95% CI) | <0.01 (<0.01, <0.01) |  |  |
|  | R squared | 0.68 |  |  |
| Relative NIR | |  |  |  |
|  | Intercept | -0.20 (-6.51, 6.12) | -0.06 | 0.95 |
|  | Body part | -5.33 (-9.52, -1.14) | -2.56 | 0.01 |
|  | PC1 | -0.84 (-1.85, 0.14) | -1.72 | 0.09 |
|  | PC2 | 0.90 (-0.35, 2.15) | 1.45 | 0.15 |
|  | PC3 | 1.29 (0.23, 2.36) | 2.74 | 0.018 |
|  | Mass | 0.23 (-2.26, 2.71) | 0.18 | 0.85 |
|  | Lambda (95% CI) | 0.65 (0.54, 0.73) |  |  |
|  | R squared | 0.33 |  |  |
| total transmission | |  |  |  |
|  | Intercept | 44.13 (26.52, 61.74) | 5.04 | <0.0001 |
|  | Body part | 13.05 (1.21, 24.89) | 2.21 | 0.03 |
|  | PC1 | 2.10 (-0.43, 4.64) | 1.67 | 0.10 |
|  | PC2 | -4.26 (-7.28, -1.25) | -2.84 | 0.007 |
|  | PC3 | -2.13 (-4.74, 0.49) | -1.64 | 0.11 |
|  | Mass | -5.54 (-12.10, 1.02) | -1.70 | 0.096 |
|  | Lambda (95% CI) | 0.83 (0.77, 1.0) |  |  |
|  | R squared | 0.34 |  |  |

Values reported are estimates over 100 trees.
